# Strength Degradation of Foamed Lightweight Soil Due to Chemical Erosion and Wet-Dry Cycle and Its Empirical Model

**DOI:** 10.3390/ma16196505

**Published:** 2023-09-30

**Authors:** Zhen Zhang, Yonggang Zhang, Guanbao Ye, Shenyi Zhang, Honghui Shen, Yonggui Chen

**Affiliations:** 1 Key Laboratory of Geotechnical and Underground Engineering of Ministry of Education, Department of Geotechnical Engineering, Tongji University, Shanghai 200092, China; zhenzhang@tongji.edu.cn (Z.Z.); demonzhangyg@163.com (Y.Z.); 1830170@tongji.edu.cn (H.S.); cyg@tongji.edu.cn (Y.C.); 2 Shanghai Baosteel New Building Materials Technology Co., Ltd., Shanghai 200942, China; zsybgjc@163.com

**Keywords:** foamed lightweight soil, chemical soaking, wet-dry cycling, coupling effect, prediction method

## Abstract

Foamed lightweight soils (FLS) have been extensively used as backfill material in the construction of transportation infrastructures. However, in the regions consisting of salt-rich soft soil, the earth structure made by FLS experiences both fluctuation of groundwater and chemical environment erosion, which would accelerate the deterioration of its long-term performance. This study conducted laboratory tests to explore the deterioration of FLS in strength after being eroded by sulfate attack and/or wet-dry cycling, where the influencing factors of FLS density, concentration of sulfate solution, and cation type (i.e., Na^+^ and Mg^2+^) were considered. An unconfined compressive test (UCT) was conducted, and the corrosion-resistant coefficient (CRC) was adopted to evaluate the erosion degree after the specimens experienced sulfate attack and/or dry-wet cycling for a certain period. The research results show that the erosion of the FLS specimen under the coupling effect of sulfate attack and dry-wet cycling was more remarkable than that only under chemical soaking, and Na_2_SO_4_ solution had a severe erosion effect as compared with MgSO_4_ solution when other conditions were kept constant. An empirical model is proposed based on the test results, and its reliability has been verified with other test results from the literature. The proposed model provides an alternative for engineers to estimate the strength deterioration of FLS on real structures in a preliminary design.

## 1. Introduction

FLS contains a large number of closed voids formed by a mixture of prefabricated foam, cement, and water, and sometimes clay or sand can be added to adjust its cost and performance [1]. Due to the adjustable density and strength of FLS, good engineering performance, and ease of construction [2], it has been increasingly used in practice, including abutment embankment, slope treatment, retaining wall backfilling, and pipeline backfilling [3,4].

The real service environment in which the FLS works is complex. The FLS will suffer rainfall infiltration, evaporation or fluctuation of the groundwater table, temperature change, freeze and thawing, salt solution erosion, and so on [5,6]. The existing studies have presented that such environmental conditions affect the long-term performance of FLS [4,7,8]. Thus, it is vital to investigate the mechanical performance of the FLS considering the real service environment. Indu and Ramamurthy [9] studied the effects of types of sulfate cation on the expansion of the FLS synthesized by two synthetic surfactants. The results showed that the FLS soaked in sodium sulfate had a higher expansion rate. Neramitkornburi et al. [10] found that the strength reduction in the FLS using fly ash and cement as cementitious materials under the dry-wet cycling was caused by the expansion and contraction of clay components, which in turn led to the development of cracks and the destruction of cementitious structures. Liu et al. [11] compared the strengths and mass change rates of cement FLS and geopolymer FLS under dry-wet cycling and found that the strength of geopolymer FLS reduced rapidly with the increasing number of dry-wet cycling and the degree of damage increased gradually, while there was no significant decrease in the strength of the cement FLS. Some studies further investigated the techniques to improve the erosion resistance of FLS. Mamun and Bindiganavile [12] studied the durability of plain and fiber-reinforced aerated FLS in sulfate solution. As the density increases, plain and fiber-reinforced aerated lightweight soil is more susceptible to sulfuric acid erosion, and the durability deteriorates with increasing immersion time. Wang et al. [13] pointed out that the addition of slag components improved the pore internal structure of FLS, further enhancing its corrosion resistance. Raj et al. [14] found the addition of polyvinyl alcohol fiber and coconut shell fiber in the FLS improved its durability than that that only with fiber. Othman et al. [15] noted that there was a certain enhancement in the resistance of FLS to sulfate corrosion when treated waste-bleaching clay was utilized as a component of FLS. Al-Shwaiter et al. [16] studied palm oil fuel ash as a substitute for sand components in FLS and revealed that the mechanical properties and microstructure of FLS were further improved with the varying contents of palm oil fuel ash. It should be noted that the above studies mainly focus on the performance of FLS under a single factor, such as chemical soaking in sulfate solution or wet-dry cycling. However, the FLS is affected by a coupling of several environmental conditions in service. In the regions consisting of salt-rich soft soil, such as coastal regions or saline-alkali areas, due to the higher salinity in groundwater and the fluctuation of the groundwater table, the earth structure made by FLS may simultaneously experience wet-dry cycles and salt solution attack during its service life. It is still a question of whether there is a coupling effect as compared with the condition under a single erosion effect.

Furthermore, it is of significance to predict the long-term strength performance of FLS in design. Liu et al. [17] combined the analytic hierarchy process with fuzzy comprehensive evaluation to establish a multi-level evaluation model for evaluating the durability of FLC in roadbed engineering. Rao et al. [18] developed a method for predicting the dynamic strength of FLS under cyclic loading. Nguyen et al. [19] predicted the compressive strength of FLS based on a deep neural network model. Zhang et al. [20] optimized the performance of FLS mixtures using a new multi-objective FA model. Pan et al. [21] developed a parameter feature extraction method based on principal component analysis and established a machine learning model for feature optimization of principal component analysis to predict the compressive strength of the FLS. However, there are few relevant strength degradation prediction models of FLS under chemical erosion and wet-dry cycles.

This paper conducted a series of tests to investigate the deterioration of mechanical properties of FLS specimens eroded by sulfate attack and wet-dry cycling. Twenty-two test groups were designed with considering the influences of FLS density (i.e., 0.8 g/cm^3^ and 0.9 g/cm^3^), SO_4_^2−^ concentration of sulfate solution (i.e., 0.338% and 3.38%), and cation type (i.e., Na^+^ and Mg^2+^). Based on the unconfined compressive test (UCT), the strength variation of the specimen was revealed. Furthermore, an empirical model was developed to predict the strength degradation of the FLS specimen under chemical erosion and wet-dry cycle. The proposed model was verified using a comparison with the reported studies.

## 2. Materials and Scheme of Test

### 2.1. Test Materials and Specimen Preparation

**Figure 1:**
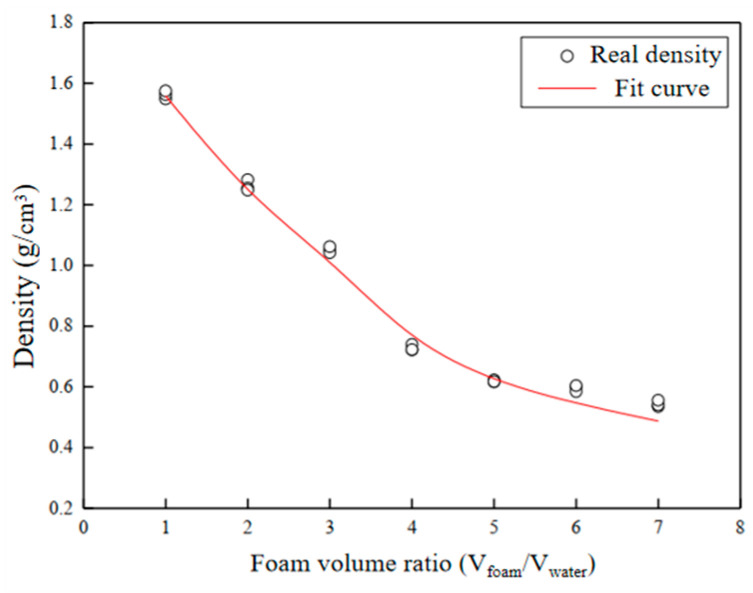
The variation curve of density of foamed lightweight soils with foam volume ratio.

The FLS in this experiment was mainly made of three components: cement, tap water, and foaming agent. In practical engineering applications, the FLS with a density of 500–1000 kg/m^3^ is usually used [4]. Therefore, specimens with densities of 800 and 900 kg/m^3^ were selected in this study. Over a series of trial tests, the relationship between foam volume ratio and density was obtained, as shown in Figure 1. It can be seen that with the increase in foam volume ratio, the density of foamed lightweight soils gradually decreased. As the target densities of the FLS specimen in this study were 800 kg/cm^3^ and 900 kg/cm^3^, the corresponding foam volume ratios were 4 and 3.5, respectively. The mix scheme of the specimen is shown in Table 1. The specimen has a diameter of 38 mm and a height of 80 mm. The preparation of the specimen referred to the Chinese industrial standard CJJ/T177 (2012) [22]. The foams were produced by mixing water, foaming agents, and compressed air together using a foam generator. Water and cement were thoroughly mixed and stirred to obtain a uniform cement slurry. The water-cement ratio was 0.45. Additionally, the prepared foams were mixed uniformly with the prepared cement slurry, and the density of the FLS was adjusted by controlling the volume of foam. The mixture of foamed cement slurry was poured into the mold. After cured for 24 h, the mold was removed and the specimens were maintained in an environment with a temperature of 25 °C and a humidity of 95% for 28 days.

### 2.2. Test Plan and Procedures

Considering a typical concentration range of sulfate solution in practice [9], the Na_2_SO_4_ solution with mass-to-mass concentrations of 0.5% and 5%, which corresponded to the SO_4_^2−^ concentration of 0.338% and 3.38%, respectively, and the MgSO_4_ solution with mass-to-mass concentration of 4.24%, were selected in this study. The MgSO_4_ solution had the same SO_4_^2−^ concentration as the 5% Na_2_SO_4_ solution. In the regions consisting of salt-rich soft soil, the FLS that are deeply under the groundwater level or the water level may be eroded by the sulfate immersion, while the FLS near the groundwater level or the water level may be eroded by both sulfate immersion and wet-dry cycling. Thus, two types of tests, i.e., sulfate immersion test and wet-dry cycle test combined with sulfate attack, were conducted. The UCT was conducted before the sulfate attack to obtain the initial strength of the specimens, which were used as reference values for analysis.

In the sulfate immersion test, the specimen was soaked in a sulfate solution (Na_2_SO_4_ or MgSO_4_) with a controlled concentration for 28 days and 56 days, respectively. For an ordinary project, the determination of properties of the FLS after 28-day and 56-day immersion is commonly required in design, while for some major projects, the immersion test for a longer period (e.g., 84 days or even 120 days) is needed. As the FLS is commonly adopted as a backfill material in roadway embankment projects, this study selected the immersion ages of 28 days and 56 days [9,11,15]. The solution should be changed once a week. After reaching the design age for immersion, the specimens were taken out for a compressive strength test. 

**Figure 2:**
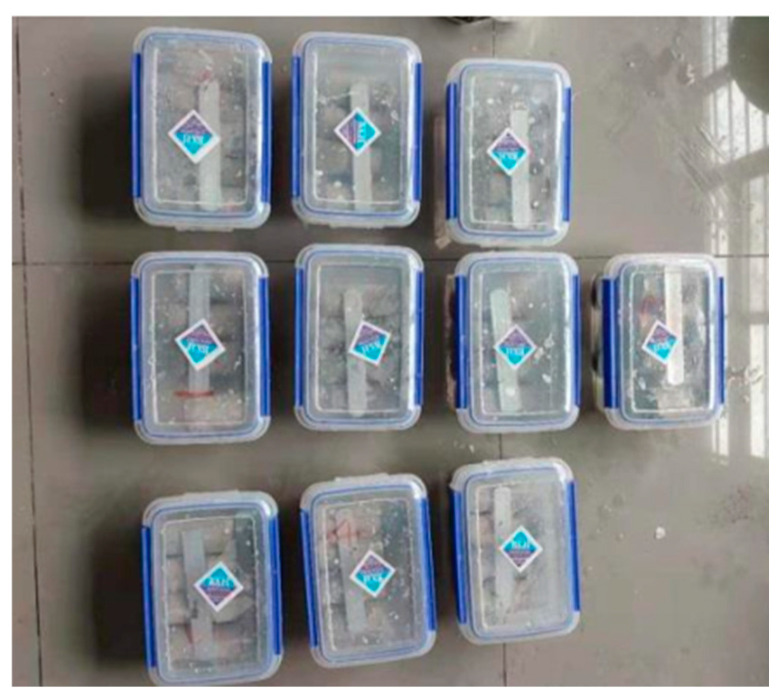
Sulfate solution immersion operation of the FLS in the immersion box.

For the wet-dry cycle test combined with sulfate attack, the specimens were placed in a prepared solution and soaked for 24 h (as shown in Figure 2). Additionally, the specimens were dried in an oven with the temperature of 40 °C for 24 h. The above test procedure was called one wet-dry cycle, and this procedure was repeated 5 cycles and 10 cycles in this study.

Table 2 summarizes the test groups in this study. Three specimens were prepared in each group. For conveniently presenting the test results, the test groups were denoted as D*x*-C*y*Na/Mg-SA/DW*z*, in which D*x* represents the specimen density *x*, C*y*Na/Mg represents the concentration *y* of the Na_2_SO_4_ or MgSO_4_ solution, SA/DW*z* represents the sulfate immersion test or the wet-dry cycle test combined with sulfate attack for a period or cycles of *z*, respectively. For example, D0.8-C0.5Na-SA28 denotes the sulfate attack test with a specimen of 0.8 g/cm^3^ in 0.5% Na_2_SO_4_ solution for 28 days. For comparison purposes, the control test groups without sulfate erosion were also conducted, which were denoted as D0.8 and D0.9. N/A indicates that no such operation has been performed.

**Figure 3:**
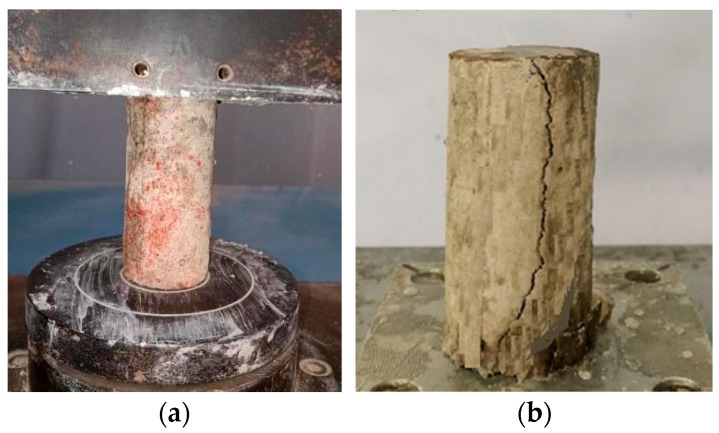
Photos during and after compression test. (**a**) Photos during compression test. (**b**) Photos during and after compression test.

After the specimens were eroded by the sulfate attack or the wet-dry cycling combined with the sulfate attack, the UCT was conducted to obtain the residual strength of the specimen. The UCT was carried out with reference to the Chinese industrial code GB/T50081(2002) [23]. During the loading phase, the loading rate of the universal testing machine was 0.2 mm/min. By recording the load at the time of specimen failure, the compressive strength of the FLS can be obtained, as shown in Figure 3.

## 3. Test Results and Analysis

### 3.1. Appearances and Mass Change

**Figure 4:**
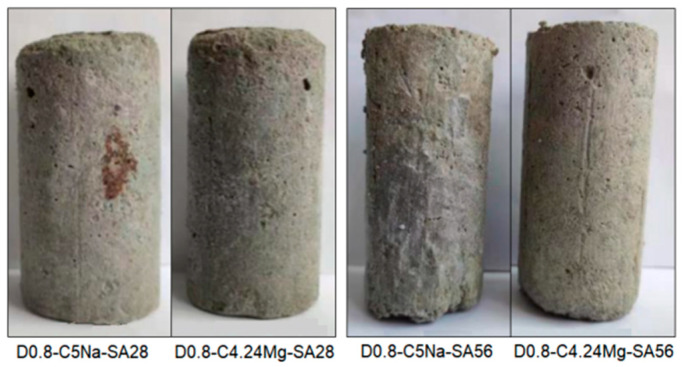

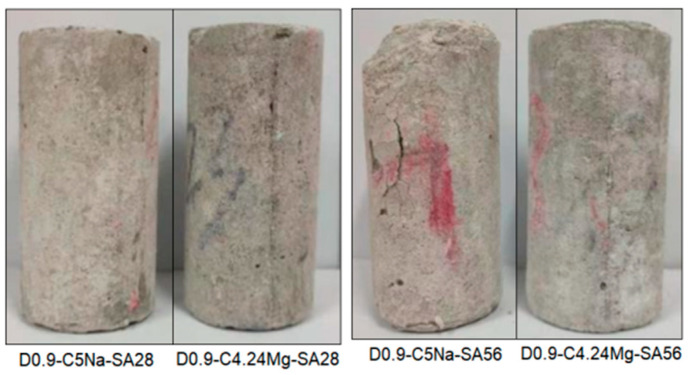
Appearances of the specimens after sulfate immersion test.

**Figure 5:**
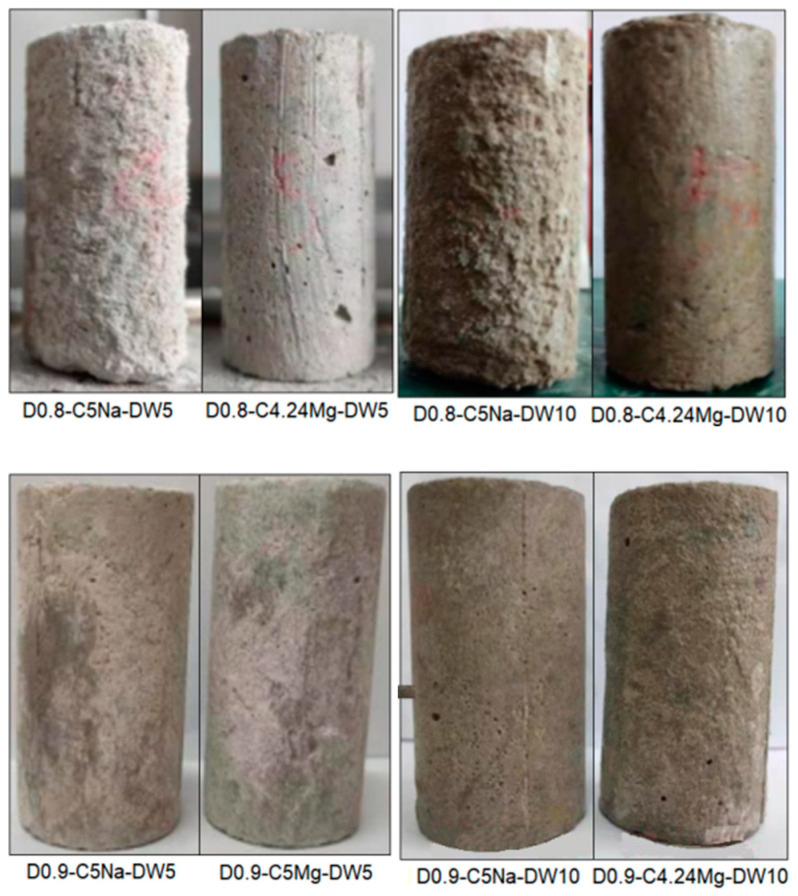
Appearances of the specimens after the wet-dry cycle test combined with sulfate attack.

Figure 4 and Figure 5 show the appearance of the specimens after erosion. It can be seen that the surfaces of the specimens were peeled, and some had cracks after erosion. The higher SO_4_^2−^ concentration of the sulfate solution (i.e., 3.38%) further intensified the corrosion of the specimens, as more spalling and cracks appeared. The main reason for this phenomenon was due to the reaction of the SO_4_^2−^ with hydration products such as calcium aluminate hydrate and calcium hydroxide to generate ettringite and gypsum [9], as well as the infiltration of the Na_2_SO_4_ solution into the specimen. Moreover, the Na_2_SO_4_ solution yielded a stronger erosion effect on the specimen as compared with the MgSO_4_ solution with the same SO_4_^2−^ concentration. This phenomenon was mainly caused by the chemical reaction between the MgSO_4_ solution and the FLS in the early stage of soaking; the generated Mg(OH)_2_ formed a protective film on the surface, preventing the infiltration of salt solution and the enrichment of hydration products in the specimen [9]. The specimens with the density of 0.9 g/cm^3^ had less spalling and cracks than the specimen of 0.8 g/cm^3^ under the same erosion condition.

**Figure 6:**
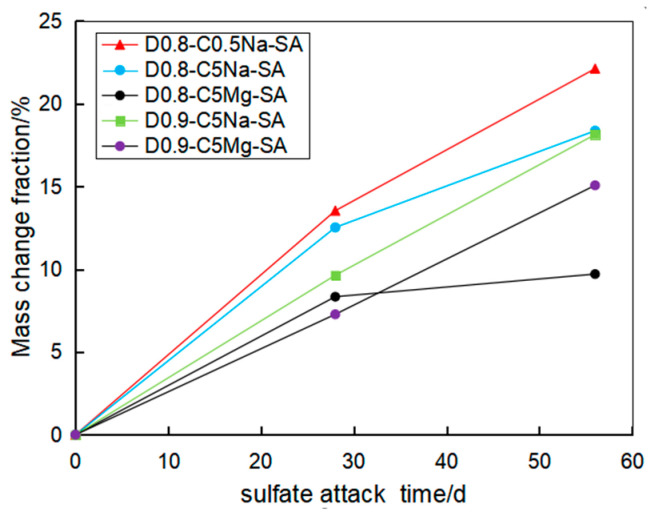
Mass change of the specimens of 0.8 and 0.9 g/cm^3^ after sulfate immersion test.

Figure 6 displays the variation of the mass change rate of the FLS with densities of 0.8 and 0.9 g/cm^3^ with the immersion time. It can be seen that the mass of the FLS increased with the sulfate attack time. Indu and Ramamurthy [9] revealed that the mass increase in the FLS was caused by the reaction of sulfate with Calcium aluminates and Calcium hydroxide to generate ettringite and gypsum. Generally, the higher the concentration, the more ettringite and gypsum were generated; however, under the erosion of chemical solution in this study, the increase in mass of the specimen immersed in 0.5% Na_2_SO_4_ solution was greater than that in 5% Na_2_SO_4_ solution. This can be explained by the fact that the specimen surface had slag falling and block missing under the sulfate attack, resulting in a certain amount of mass loss (see Figure 5). Under the erosion of the same sulfate concentration, the mass increase in the 4.24% MgSO_4_ solution was less than that in the 5% Na_2_SO_4_ solution. Indu and Ramamurthy [9] indicated that the MgSO_4_ solution reacted with the FLS to generate Mg(OH)_2_ in the early stage of chemical immersion, forming a protective film on the surface, which prevented the infiltration of sulfate solution and the enrichment of hydration products in the specimen. By comparing the specimens of 0.8 g/cm^3^ and 0.9 g/cm^3^, it can be seen that the specimen with 0.9 g/cm^3^ exhibited relatively smaller mass growth than the specimen with 0.8 g/cm^3^ under the same condition. This indicates that the higher the density of the FLS, the stronger its ability to resist chemical solution erosion.

**Figure 7:**
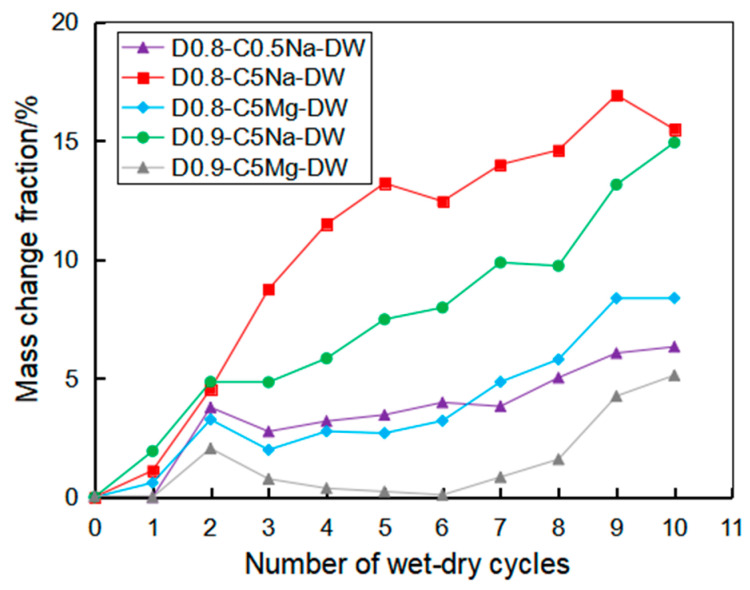
Mass change of the specimens of 0.8 and 0.9 g/cm^3^ after the wet-dry cycle test combined with sulfate attack.

Figure 7 presents the variation of the mass change rate of the specimens under the coupling effect of chemical erosion and wet-dry cycle. The observations of the mass change were similar to those under the sulfate immersion. With the increase in the wet-dry cycles, the mass rate of the specimen gradually increased. The specimen in the 5% Na_2_SO_4_ solution led to a much more significant mass increase than that in the 0.5% Na_2_SO_4_ solution. By comparing the specimens of 0.8 and 0.9 g/cm^3^, the change in mass of the specimen with 0.9 g/cm^3^ was relatively small, indicating that the FLS with high density had a stronger ability to resist the coupling effect of chemical erosion and wet-dry cycle.

### 3.2. Stress vs. Strain Curve

**Figure 8:**
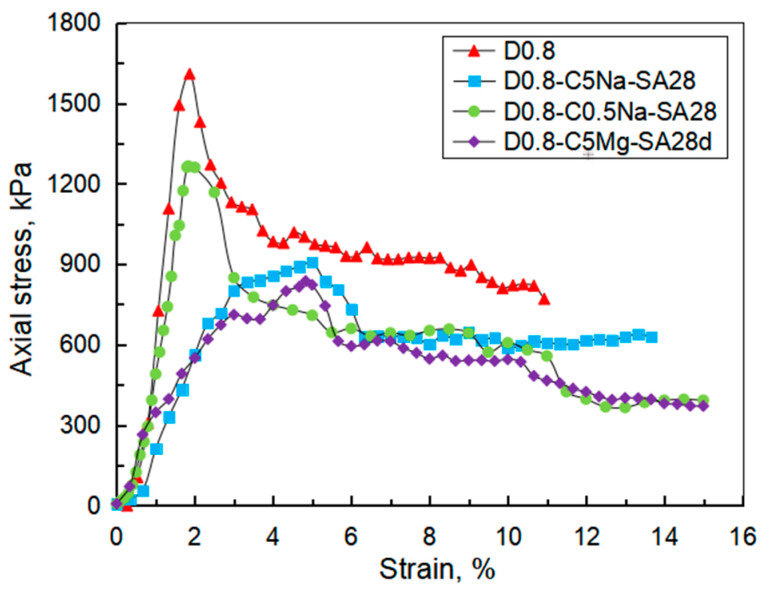
Stress–strain curves of the specimens of 0.8 g/cm^3^ under sulfate immersion for 28 days.

As the stress–strain curves of FLS obtained from the UCT were similar, only the stress–strain curves of the specimens of 0.8 g/cm^3^ under 28-day sulfate immersion are shown in Figure 8. As the strain increased, the stress also rapidly increased in the initial stage and quickly reached its peak (i.e., also called unconfined compressive strength). Additionally, the stress decreased rapidly and stabilized at a constant value which is called residual strength.

**Figure 9:**
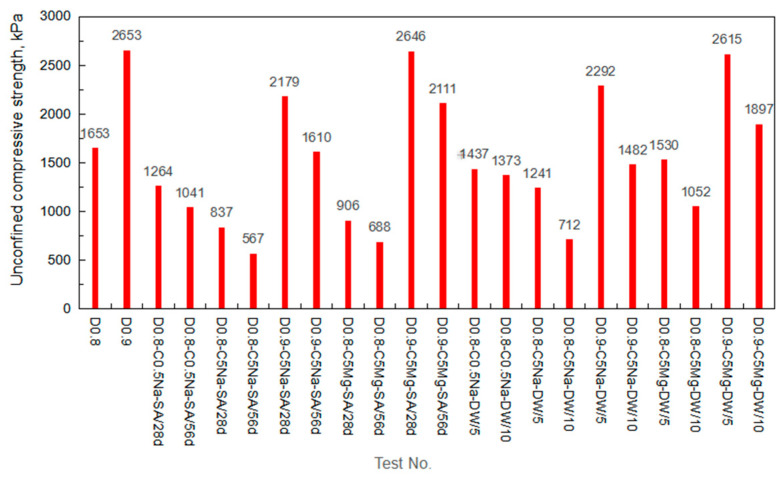
Unconfined compressive strength of the specimens.

Figure 8 also proves that the erosion condition significantly impacted the specimen strength. For the specimens soaked in the solutions of 0.5% Na_2_SO_4_, 5% Na_2_SO_4,_ and 4.24% MgSO_4_, the unconfined compressive strengths were 1.26, 0.84, and 0.91 MPa, respectively. As compared with the specimen without the sulfate immersion, the unconfined compressive strength of the specimens after soaked by the three sulfate solutions decreased by 23.5%, 49.38%, and 45.2%, respectively. The sulfate solution with higher concentration had a greater impact on the reduction in the specimen strength. In addition, the Na_2_SO_4_ solution exhibited greater influence on the deterioration of the specimen strength than the MgSO_4_ solution with the same SO_4_^2−^ concentration. Figure 9 summarizes the unconfined compressive strength of the specimens under different test conditions. Clearly, the unconfined compressive strength of the specimens decreased with the increasing immersion age or wet-dry cycles. The corrosion resistance of the FLS specimen under sulfate attack will be analyzed in the next section.

### 3.3. Corrosion Resistance of the Specimen under Sulfate Attack

To evaluate the corrosion resistance of the specimen due to the sulfate attack, the corrosion resistant coefficient (CRC) is introduced, which is defined as the residual unconfined compressive strength of the specimen after sulfate solution erosion divided by its initial unconfined compressive strength. The value of the corrosion-resistant coefficient is between 0 and 100%, while the value close to 100% indicates a stronger corrosion resistance.

(1) $$\mathrm{CRC}=\frac{P_{r}}{P_{i}}\times 100\%$$

where CRC is the corrosion-resistant coefficient, *P*_r_ is the remaining unconfined compressive strength of the specimen after sulfate solution erosion, and *P*_i_ is the initial unconfined compressive strength.

**Figure 10:**
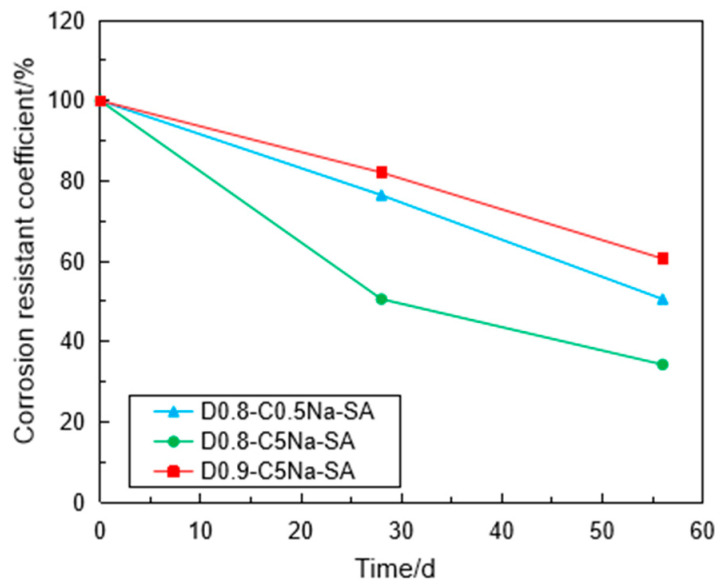
Variation of CRC of the specimen with the immersion time.

Figure 10 shows the variation of the CRC with the immersion time in the Na_2_SO_4_ solution. The CRC values were reduced with the immersion time regardless of the concentration of the Na_2_SO_4_ solution and the density of the specimen. It is clear that the higher concentration of the Na_2_SO_4_ solution had a significant influence on the strength reduction in the specimen when the densities of the specimen were the same. The CRC of the specimen of 0.9 g/cm^3^ was 60.7% after sulfate attack for 56 days, while the CRC of the specimen of 0.8 g/cm^3^ was 34.3% under the same immersion condition. This indicates that the high-density FLS had stronger erosion resistance under the same Na_2_SO_4_ concentration. This can be explained by the fact that high-density FLS specimens had strong cementation structures due to the high content of cement matrix [15].

**Figure 11:**
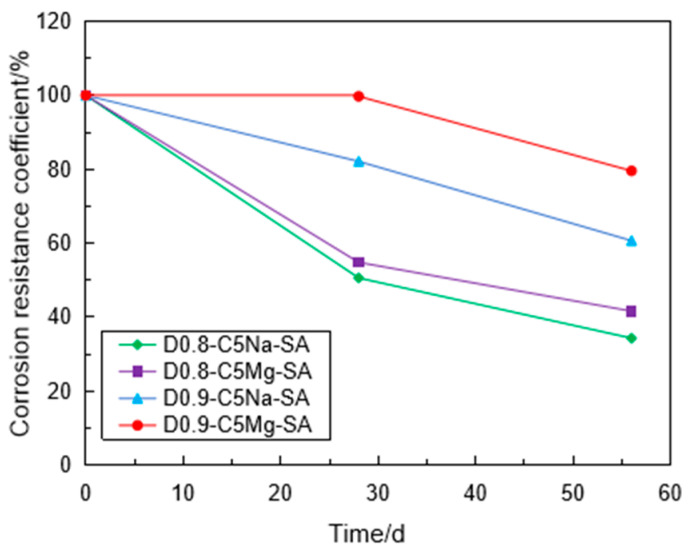
Variation of CRC of the specimen with the immersion time in the different sulfate solutions.

Figure 11 shows the variation of the CRC with the immersion time in different sulfate solutions. The CRC values of the specimens with 0.8 g/cm^3^ after sulfate immersion in 5% Na_2_SO_4_ solution were 51% and 34.3% for 28 days and 56 days, respectively, while the CRC values of the specimens in 4.24% MgSO_4_ solution were 54.8% and 41.6%, respectively. For the sulfate solution with the same SO_4_^2−^ concentration, the FLS specimen was eroded more seriously in the Na_2_SO_4_ solution than in the MgSO_4_ solution. This phenomenon was more obvious for the specimen with 0.9 g/cm^3^. When the FLS specimen was immersed in the MgSO_4_ solution, a film of Mg(OH)_2_ was produced on the surface of the specimen, preventing the specimen from further erosion [9].

### 3.4. Corrosion Resistance of the Specimen under Coupling Effect

**Figure 12:**
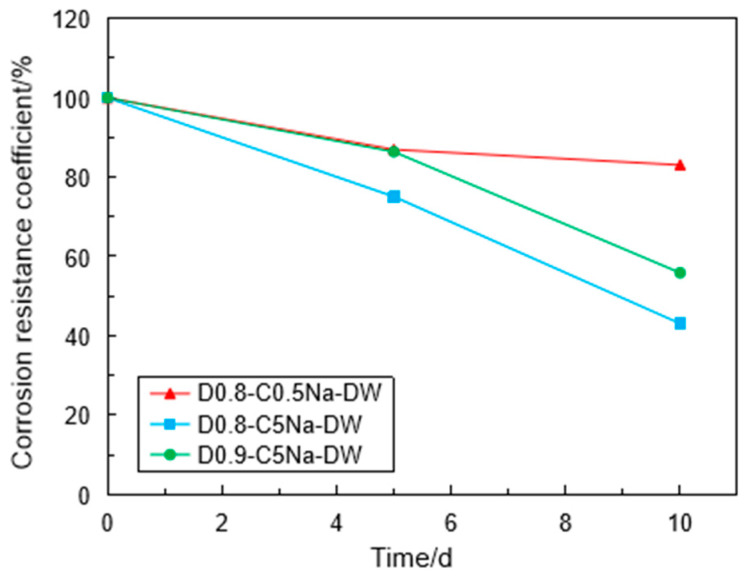
Variation of CRC of the specimen with the wet-dry cycles.

**Figure 13:**
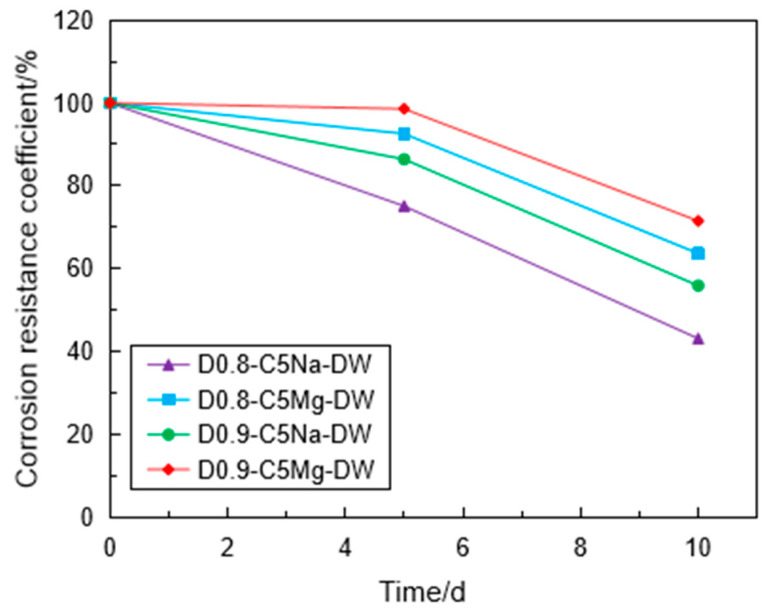
Variation of CRC of the specimen in the different sulfate solutions with the wet-dry cycles.

Figure 12 shows the variation of the CRC under the coupling effect in the Na_2_SO_4_ solution. The CRC values reduced with the increase in the wet-dry cycles regardless of solution concentration and specimen density. For the specimen with 0.8 g/cm^3^ after 10 cycles, the CRC was 43.1% in the 5% Na_2_SO_4_ solution, while the CRC was 83.1% in the 0.5% Na_2_SO_4_ solution. As expected, the erosion of the FLS specimen is more serious in the sulfate solution of high concentration under the coupling effect. Compared with the specimen of 0.8 g/cm^3^, the CRC of the specimen of 0.9 g/cm^3^ reduced slowly, indicating that the high-density FLS specimen had strong erosion resistance against the coupling effect when other conditions were kept constant. Figure 13 shows the variation of the CRC with the dry-wet cycles in the different cation types (i.e., Na^+^ and Mg^2+^). Under the coupling effect, the CRC of the FLS specimen in the Na_2_SO_4_ solution reduced significantly than that in the MgSO_4_ solution with the increase in dry-wet cycles.

### 3.5. Comparison of Sulfate Immersion and Coupling Effect

**Figure 14:**
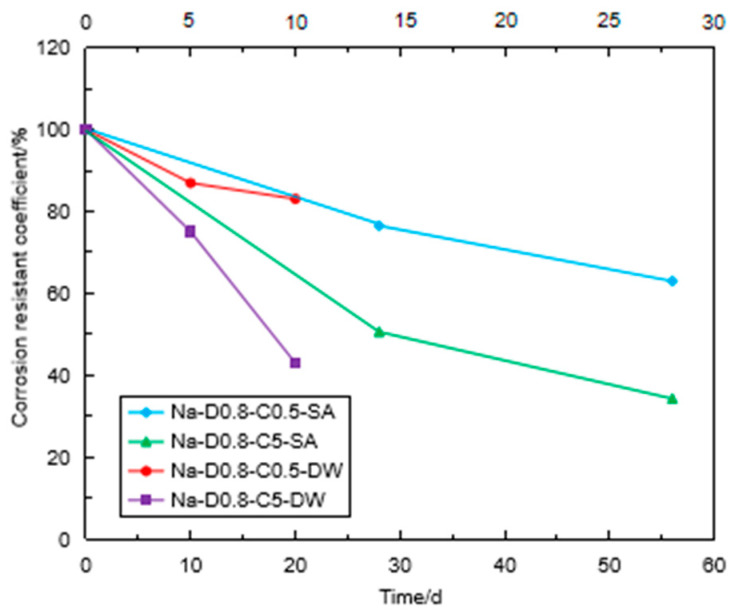
Comparison of the CRC under sulfate immersion and coupling effect.

The former section confirmed that the strength of the FLS specimen was reduced due to the sulfate immersion and the coupling effect of chemical erosion and the wet-dry cycle. To compare the influence of these two erosion conditions, Figure 14 shows the CRC of the FLS specimens under two erosion conditions. Eroded by the Na_2_SO_4_ solution with the same concentration, the coupling effect accelerated the reduction in the specimen strength. In the 0.5% Na_2_SO_4_ solution, the CRC curve of the specimen under the coupling effect was slightly below those under the sulfate immersion. However, when the specimens were eroded by the 5% Na_2_SO_4_ solution, the CRC of the specimen under the coupling effect decreased faster than that under the sulfate immersion, and the gap was further widened with the increase in the erosion age. From a practical point of view, more attention must be paid when the backfilled FLS faced both the chemical erosion and wet-dry cycle, as the strength reduction in FLS due to the coupling effect was more significant.

## 4. Empirical Method for Predicting the CRC of FLS

### 4.1. Strength Degradation Model

It is confirmed that the strength of the FLS decreased with erosion age due to the sulfate attack regardless of the solution concentration and sulfate type. Other studies also revealed similar results [9,11,24]. Thus, an exponential function was selected to build the strength degradation model,

(2) $$R_{t}=\lambda R_{0}e^{-\beta T}$$

where *R*_0_ is the initial compressive strength of FLS without sulfate attack; *R*_t_ is the compressive strength of FLS after eroded by sulfate solution for a period of *T*; *λ* and *β* are undetermined coefficients related to the influencing factors of concentration and type of sulfate solution, density of FLS, and so on. As *CRC* equals to *R*_t_/*R*_0_, Equation (2) can be rewritten as:

(3) $$CRC=\lambda e^{-\beta T}$$

The influencing factors, including the concentration and type of sulfate solution, as well as the density of FLS, were discussed in this study. Based on the variable separation method, Equation (2) is rebuilt in the following form:

(4) $$CRC=\lambda e^{-\beta_{1}(c)\beta_{2}(p)T}$$

where *β*_1_(*c*) and *β*_2_(*ρ*) are the functions related to the concentration of sulfate solution and density of FLS, respectively; *λ* is the factor considering the type of sulfate solution.

### 4.2. Determination and Verification of the Proposed Model

The multivariable modeling was adopted to determine the proposed model based on the test results. The average fitting results of Equations (5) and (6) were 4% and 2% higher than the test values (Figure 14), respectively, further demonstrating the high confidence of the proposed prediction model for strength prediction of the FLS. The proposed model is presented as follows:

(5) $$CRC=\lambda e^{-0.0008R_{c}R_{\rho }{}^{-9.6}T}$$

**Figure 15:**
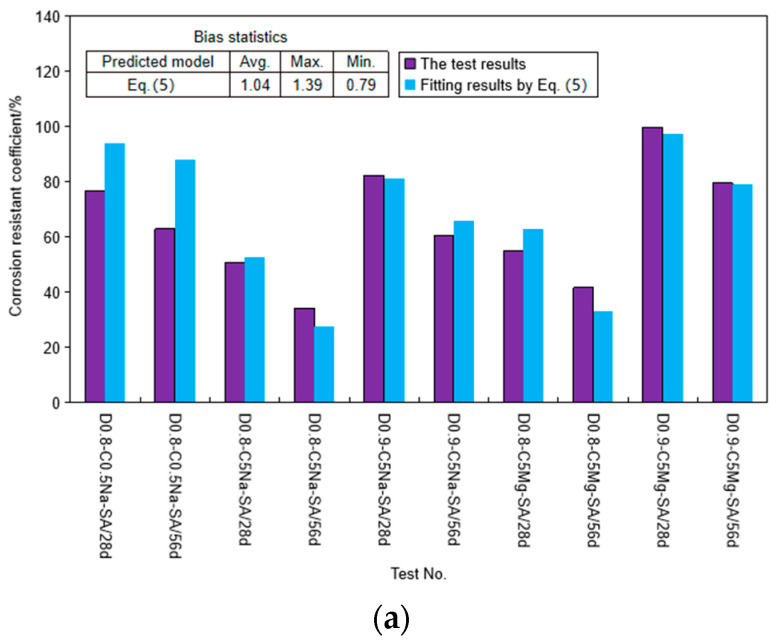

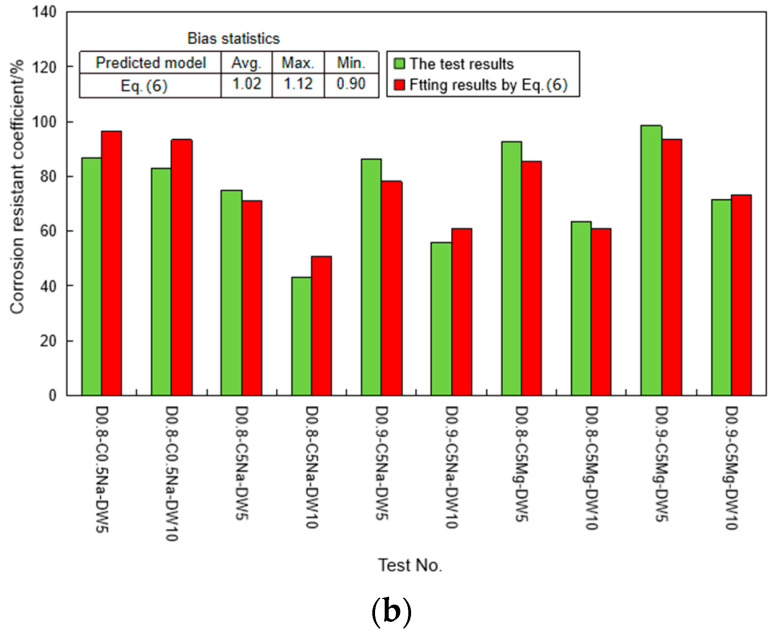
Test and fitting results: (**a**) under sulfate immersion; (**b**) under coupling effect of chemical attack and wet-dry cycle.

Under the coupling effect of sulfate attack and wet-dry cycle,

(6) $$CRC=\lambda e^{-0.0011R_{c}R_{\rho }{}^{-2.7}T}$$

where *R_c_* is the concentration ratio of SO_4_^2−^ solution (%); and *R_ρ_* is the relative density of FLS, which is the FLS density divided by the density of water; *λ* is 1.0 and 1.2 for Na_2_SO_4_ solution and MgSO_4_ solution, respectively. *T* equals the days of sulfate immersion ages when the FLS is under sulfate immersion or equals the days equivalent to the wet-dry cycles. The average fitting results of Equations (5) and (6) were 4% and 2% higher than the test values (Figure 15), respectively, demonstrating the good agreement of the proposed prediction model.

**Figure 16:**
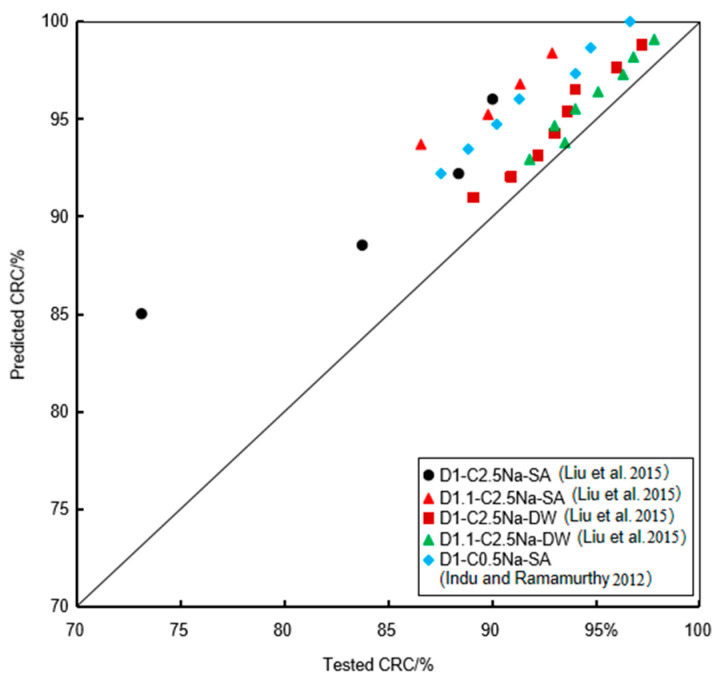
The comparison of the test results and predicted results under sulfate immersion and coupling effect of chemical attack and wet-dry cycle.

The results from the literature were selected to verify the proposed empirical model. Liu et al. [11] conducted a series of tests on the FLS specimens with the densities of 1 g/cm^3^ and 1.1 g/cm^3^ eroded by 2.5% Na_2_SO_4_ solution for 120 days, as well as a coupling erosion of chemical attack and wet-dry cycle. Indu and Ramamurthy [9] conducted a series of tests with the FLS specimens with densities of 1 g/cm^3^ eroded by 0.5% Na_2_SO_4_ solution for 350 days. Figure 16 shows the comparison of the tested CRC and the corresponding predicted CRC. It can be seen that the tested CRC and the predicted CRC were near a 45° straight line, showing a reasonable agreement with the test results, either by sulfate immersion or by coupling effect of chemical attack and wet-dry cycle.

### 4.3. Sensitivity Analysis of Influencing Factors

To quantitatively characterize the sensitivity of the influence factors (i.e., SO_4_^2−^ concentration and FLS density) on strength degradation of FLS, the orthogonal experiments and range analysis were conducted in this section [25]. The density of the FLS generally ranges from 0.6 to 1.6 g/cm^3^ in practice [4,26]. The SO_4_^2−^ concentration in the underground water near the coastal region is commonly within the range of 0.338% to 3.38% [27]. Table 3 shows the orthogonal experimental tables for the influencing factors of SO_4_^2−^ concentration and FLS density. Each influencing factor was divided into three levels, labeled A, B, and C. The SO_4_^2−^ concentration was set at three concentrations, namely, A = 0.338%, B = 1.69%, and C = 3.38%, respectively. Similarly, the densities were set at levels of A = 0.6 g/cm^3^, B = 1.1 g/cm^3^, and C = 1.6 g/cm^3^, respectively. The proposed empirical model was adopted to predict the CRC of FLS after one year. 

Under sulfate immersion, the range values of SO_4_^2−^ concentration and FLS density were 0.096 and 0.995, respectively. This indicated that the FLS density had a more significant influence than the CRC of FLS as compared with SO_4_^2−^ concentration under sulfate immersion. The range values of SO_4_^2−^ concentration and FLS density were 0.429 and 0.430, respectively, under the coupling effect of chemical attack and wet-dry cycle. The impact of concentration and density on CRC became similar under the coupling effect of chemical attack and wet-dry cycle. The strength degradation was sensitive to the FLS density. The FLS density needs to be carefully selected when the FLS would suffer a sulfate attack from a practical point of view.

### 4.4. Discussion and Limitation

Based on the test results, under sulfate immersion, the sulfate attack had a greater impact on the reduction in the specimen strength; under coupling effect of chemical attack and wet-dry cycle, the erosion of the FLS specimen is more seriously in the sulfate solution with high concentration, the FLS specimen with high density has strong erosion resistance. Other research results [9,11,24] have also shown that high concentrations of sodium sulfate have a stronger corrosiveness to the FLS than low concentrations of sodium sulfate. The Na_2_SO_4_ solution had a serious erosion effect as compared with the MgSO_4_ solution when other conditions were kept constant, indicating that sodium sulfate has stronger corrosiveness. Moreover, the erosion of the FLS specimen under the coupling effect is more significant than that only under chemical soaking.

Taking into account factors of FLS density, sulfate solution concentration, and sulfate solution type, an empirical method was proposed to predict the strength degradation of FLS under sulfate immersion or under the coupling effect of sulfate attack and wet-dry cycle. Although the comparison with the results from Indu and Ramamurthy [9] and Liu et al. [11] shows good agreement, due to the size effect of the specimen in this study, further verification is necessary to see whether the test results can be extrapolated for practical use on real structures.

In addition, the FLS in the actual application could differ from the FLS in this experiment. The FLS sometimes adds some additives, such as fly ash, dredging soil, olive oil industrial waste, soft clay, etc. [28,29,30,31,32,33,34], to improve its various properties. Thus, there may be deviations in the predictions when using this empirical method in practice. It is noted that chemical erosion and wet-dry cycle tests on real structures are always time-consuming and costly. The proposed model provides an alternative for engineers to roughly estimate the strength deterioration of FLS on real structures in a preliminary design.

## 5. Conclusions

This study conducted a series of tests to investigate the strength deterioration of FLS specimens eroded by sulfate attack and wet-dry cycling. Based on the test results and analyses, the following conclusions can be drawn:

(1) The mass of the FLS increased with the sulfate attack time caused by the reaction of sulfate with Calcium aluminates and Calcium hydroxide to generate ettringite and gypsum. Under the erosion of the same sulfate concentration, the increase in mass caused by the MgSO_4_ solution was less than that by the Na_2_SO_4_ solution with the same SO_4_^2−^ concentration. The specimen with 0.9 g/cm^3^ had relatively smaller mass growth than the specimen with 0.8 g/cm^3^ under the same condition.
(2) Using the UCT, it can be found that the sulfate attack had a greater impact on the reduction in the specimen strength. For chemical attack, the FLS specimen underwent more serious erosion in the high-concentration sulfate solution, and the high-density FLS specimen had strong erosion resistance. The Na_2_SO_4_ solution provided a more severe erosion effect compared to the MgSO_4_ solution when other conditions were kept constant. However, the erosion of the FLS specimen under the coupling effect of sulfate attack and dry-wet cycling was more significant than that only under chemical soaking.
(3) An empirical model was developed based on the test results to predict the strength degradation of FLS under sulfate immersion and under the coupling effect of chemical attack and wet-dry cycle, and it was further verified using a comparison with the reported studies. The sensitivity analysis indicated that the strength degradation was sensitive to the FLS density.

## Figures and Tables

**Table 1:** The mix scheme of the FLS specimen.

Density of the FLS (g/cm^3^)	Water-Cement Ratio	Cement Quality (kg)	Water Quality (kg)	Foam Volume (L)	Foam Volume Ratio (V_foam_/V_water_)
800	0.45	4.4	2	8	4
900	0.45	4.4	2	7	3.5

**Table 2:** Test program.

Test No.	*ρ*/g/cm^3^	*m*	*c*/%	*p*/d	*N*
D0.8	0.8	N/A	N/A	N/A	N/A
D0.9	0.9	N/A	N/A	N/A	N/A
D0.8-C0.5Na-SA28/56	0.8	Na_2_SO_4_	0.5	28, 56	0
D0.8-C5Na-SA28/56	0.8	Na_2_SO_4_	5.0	28, 56	0
D0.8-C4.24Mg-SA28/56	0.8	MgSO_4_	4.24	28, 56	0
D0.9-C5Na-SA28/56	0.9	Na_2_SO_4_	5.0	28, 56	0
D0.9-C4.24Mg-SA28/56	0.9	MgSO_4_	5.0	28, 56	0
D0.8-C0.5Na-DW5/10	0.8	Na_2_SO_4_	0.5	N/A	5, 10
D0.8-C5Na-DW5/10	0.8	Na_2_SO_4_	5.0	N/A	5, 10
D0.8-C4.24Mg-DW5/10	0.8	MgSO_4_	4.24	N/A	5, 10
D0.9-C5Na-DW5/10	0.9	Na_2_SO_4_	5.0	N/A	5, 10
D0.9-C4.24Mg-DW5/10	0.9	MgSO_4_	4.24	N/A	5, 10

Note: *ρ* is the specimen density; *m* is the sulfate medium; *c* is the concentration of the sulfate solution; *p* is the soaking age; *N* is the number of dry-wet cycles; N/A indicates that no such operation has been performed.

**Table 3:** Orthogonal test and range analysis results.

Test No.	*c*/%	*ρ*/g/cm^3^	CRC Prediction	
			Equation (5)	Equation (6)
1	A(0.338)	A(0.6)	0	0.068
2	A(0.338)	B(1.1)	0.963	0.592
3	A(0.338)	C(1.6)	0.999	0.826
4	B(1.69)	A(0.6)	0	0
5	B(1.69)	B(1.1)	0.827	0.073
6	B(1.69)	C(1.6)	0.995	0.385
7	C(3.38)	A(0.6)	0	0
8	C(3.38)	B(1.1)	0.685	0.050
9	C(3.38)	C(1.6)	0.990	0.148
*Rs*	0.096	0.995	N/A	N/A
*Rc*	0.429	0.430	N/A	N/A

Note: *Rs* and *Rc* represent the range values under sulfate immersion and under coupling effect of chemical attack and wet-dry cycle, respectively. N/A indicates no numerical value.

## Data Availability

The data that support the findings of this study are available from the corresponding author, Guanbao Ye, upon reasonable request.
